# Outcomes of Different Lifestyle Approaches in a Multicentre, Open-Label, Parallel-Group, Randomised Controlled Trial of the Effectiveness of Integrating a Pragmatic Pathway for Prescribing Liraglutide 3.0 mg in Weight Management Services (STRIVE Study)

**DOI:** 10.3390/metabo15060398

**Published:** 2025-06-13

**Authors:** Werd Al-Najim, Babak Dehestani, Ahmed W. Al-Humadi, Danielle H. Bodicoat, Dimitris Papamargaritis, Michael Lean, Barbara McGowan, David R. Webb, John PH Wilding, Melanie J. Davies, Carel W. le Roux

**Affiliations:** 1 Clinical Research Centre, St. Vincent’s University Hospital, Elm Park, Dublin 4, Ireland; werd.al-najim@ucd.ie (W.A.-N.); dehestani.babak@gmail.com (B.D.); a.alhumadi@svph.ie (A.W.A.-H.); 2 Diabetes Complications Research Centre, Conway Institute, University College Dublin, Dublin 4, Ireland; 3 Independent Researcher, Leicester LE1 7RH, UK; danielle@studio3research.com; 4 Diabetes Research Centre, Leicester General Hospital, College of Life Sciences, University of Leicester, Leicester LE5 4PW, UK; dp421@leicester.ac.uk (D.P.); david.webb@uhl-tr.nhs.uk (D.R.W.); melanie.davies@uhl-tr.nhs.uk (M.J.D.); 5 National Institute for Health and Care Research (NIHR), Leicester Biomedical Research Centre, University Hospitals of Leicester NHS Trust and the University of Leicester, Leicester LE5 4PW, UK; 6 Department of Diabetes and Endocrinology, Kettering General Hospital, University Hospitals of Northamptonshire NHS Group, Kettering NN16 8UZ, UK; 7 School of Medicine, Dentistry and Nursing, University of Glasgow, Glasgow G31 2ER, UK; mike.lean@glasgow.ac.uk; 8 Diabetes, Endocrinology and Obesity (DEO) Clinical Academic Partnership, King’s Health Partners, Guy’s & St Thomas’ Hospital, London SE1 7EH, UK; barbara.mcgowan@gstt.nhs.uk; 9 Department of Cardiovascular and Metabolic Medicine, Institute of Life Course and Medical Sciences, University of Liverpool, Liverpool L7 8TX, UK; jwilding@liverpool.ac.uk

**Keywords:** obesity, lifestyle intervention, STRIVE study, severe complex obesity, specialist weight management services

## Abstract

**Background/Objectives:** The STRIVE study was a multicentre, open-label, real-world clinical trial evaluating the effectiveness of a targeted prescribing pathway for liraglutide 3.0 mg as an adjunct to standard care versus standard care alone in people with obesity attending Specialist Weight Management Services (SWMS) in the UK and Ireland. This post hoc analysis focuses on the standard care arm to explore differences in outcomes between sites, particularly the potential impact of offering meal replacements as part of usual care. **Methods:** Participants included individuals with a BMI ≥ 35 kg/m² and at least one obesity-related complication who received standard care at five SWMS sites. All sites provided specialist nutrition and exercise counselling; however, only the Dublin site (n = 40) included meal replacements as part of routine care. Baseline characteristics and weight change data were compared between the Dublin and UK cohorts (n = 92) at 52 and 104 weeks. Statistical comparisons were made using appropriate parametric and non-parametric tests. **Results:** At baseline, the Dublin cohort was significantly older (*p* < 0.01), had a higher prevalence of hypertension (*p* < 0.05), and a lower reported incidence of depression/anxiety (*p* < 0.05) than the UK cohort. At week 52, the Dublin group achieved greater mean weight loss (−6.1%, SD ± 5.7%) compared to the UK cohort (−1.3%, SD ± 6.7%, n = 27, *p* < 0.01). By week 104, Dublin participants maintained a mean weight loss of −4.4% (SD ± 5.7%) while UK participants had a mean weight gain of 0.37% (SD ± 7.6%) (*p* < 0.05). **Conclusions:** The integration of meal replacements as part of usual care may have contributed to the greater and sustained weight loss observed in the Dublin cohort compared to other SWMS in the UK.

## 1. Introduction

Patients with a body mass index (BMI) > 35 kg/m^2^ and one or more obesity-related complications can be defined as having severe and complex obesity (SCO) [1]. Such complications may include pre-diabetes, type 2 diabetes (T2DM), sleep apnoea, and hypertension (HTN) [2]. The prevalence of obesity continues to increase [3], and the rates of obesity-related complications and SCO continue to rise with it [3,4]. In Ireland and the UK, tertiary management of patients living with SCO occurs within a multidisciplinary setting within specialist weight management services (SWMSs), with the purpose of considering pharmacological and surgical options [1,2]. While these treatment options have shown great success in improving health outcomes, some barriers remain in place for their regular use, including the price and availability of medications and surgery [5,6]. Another barrier to care is that not all patients will respond to medication and surgery, but this remains difficult to predict prior to starting the interventions [7]. Finally, gastrointestinal side effects with medications and surgery are normally mild to moderate, but some patients still find these difficult to tolerate [8,9].

Lifestyle interventions are an important option for improving quality of life and health gains for people with SCO. There is no universal approach to the use of lifestyle intervention for improving health gains. General guidance for intensive lifestyle intervention has been proposed by the American Heart Association, the American College of Cardiology, and the Obesity Society [10]. Within these guidelines, the three pillars of dietary modification, physical therapy, and behavioural therapy are described [10]. However, across different countries and respective cultural contexts, the feasibility of implementing these guidelines [10] may be challenging. For example, individuals receiving high-intensity intervention are to receive a minimum of 14 in-person individual or group sessions for the first 6 months [10]. Implementing these best-practice guidelines may be difficult in more resource-challenged environments. As such, adaptability to local populations and services is an important factor in optimising lifestyle intervention for people living with obesity.

The STRIVE study was an open label, real-world, randomised controlled trial (RCT) assessing the clinical effectiveness at 52 and 104 weeks of a targeted prescribing pathway for liraglutide 3.0 mg as an adjunct to standard care in specialist weight management services (SWMSs), aiming to achieve ≥15% weight loss (WL) [2]. The STRIVE study demonstrated that 21% (54 out of 260) of the participants randomised to the targeted prescribing pathway for liraglutide 3 mg achieved more than 15% WL at one year (after passing all the three stopping rules), and approximately half of those who passed all the stopping rules and stayed on liraglutide for the second year were able to maintain ≥10% WL at 104 weeks [2,11]. Improvements in cardiometabolic risk factors and in some parameters of quality of life during the study were confirmed without new safety signals.

Our study is a post hoc analysis of the standard-care arm of the STRIVE study [11]. The aim of this analysis was to examine the differences in WL and health gain achievement within SWMSs across the UK and Irish sites involved in the STRIVE study.

## 2. Materials and Methods

The STRIVE study [2] recruited patients from five different specialist weight management centres: Dublin, Glasgow, Leicester, Liverpool, and London. Participants meeting the inclusion criteria for the trial [2] were randomised in a 2:1 manner, with 2 participants receiving liraglutide 3.0 mg in addition to standard care for every 1 participant receiving standard care alone. Randomisation occurred by way of a validated online system (sealedenvelope.com), and was stratified according to location and a BMI ≥ or ≤45 kg/m^2^. Participants were informed of their randomisation outcome at the time of randomisation.

Standard-care practice was multidisciplinary, but centres were allowed to have variations in team composition depending on local service needs and funding. Typical team members included clinical specialist nurses, dieticians, specialist physicians, psychologists, and physiotherapists. A specialist physician was available at all sites. The trial participants received best-practice care within their local centre and ongoing support throughout the trial duration of 104 weeks.

All sites delivered lifestyle interventions focused on dietary modification with a reduction of 500 kcal, physical activity for 150 min/week, and behavioural support, although the format of delivery varied. WL interventions largely focused on tailored guidance on the reduction of energy intake and promotion of physical activity. The Dublin cohort uniquely received structured partial meal replacements (Herbalife^®^) for breakfast and lunch as part of usual care, as well as supervised or supported exercise regimens. Local practices varied across sites, given that the STRIVE study was a pragmatic real-world study [2]. Monitoring and clinical visits occurred at pre-defined intervals, as previously described [2].

This is a post hoc analysis of the standard-care arm of the STRIVE trial. The detailed statistical analysis plan for the STRIVE study has been previously described [2], and the same methodology was followed for this post hoc analysis for all sites within the standard-care arm of the STRIVE Study. We set out to compare the achievement of ≥15% WL at weeks 52 and 104 between all sites and key secondary outcomes. These outcomes were as follows:

• The difference in absolute weight change between cohorts, in kg;
• Changes to parameters relating to metabolic health: the waist circumference difference from baseline (cm) and glycaemic control (HbA1c);
• Changes to core quality-of-life measures on EQ5D: self-reported anxiety/depression, the ability to carry out activities of daily living/self-care, and the ability to carry out usual activities.
• Improvements in obesity-related complications: diabetes onset, HTN, chronic pain, and mobility.

Descriptive statistics were used to summarise baseline characteristics and outcomes at weeks 52 and 104. Between-group comparisons for continuous variables (e.g., weight, HbA1c, waist circumference) were assessed using independent-samples t-tests or Mann–Whitney U tests, depending on the data distribution. Categorical variables (e.g., proportions achieving > 5%, >10%, or >15% WL) were analysed using chi-square tests or Fisher’s exact tests, as appropriate. Within-group changes from baseline were evaluated using paired t-tests. All statistical tests were two-tailed, with significance defined as *p* < 0.05. No adjustments were made for confounding variables, as this was an exploratory post hoc analysis.

## 3. Results

There were no statistically significant differences between the WL responses of the UK sites, but the WL was greater in the Dublin cohort. Thus, the UK sites were collapsed together and compared as one cohort with the Dublin cohort.

### 3.1. Participants’ Baseline Characteristics

A complete data set for 132 participants in the standard-care arm was available for analysis. There were 40 participants receiving standard care in the Dublin cohort. The remaining 92 were spread across sites based in the UK. The participant distribution in the UK cohort was as follows: Glasgow (n = 9), Leicester (n = 30), Liverpool (n = 26), and London (n = 27). The ratio of males to females was similar across all sites. Table 1 summarises the categorical baseline characteristics. Greater ethnic heterogeneity was observed within the UK cohort (*p* < 0.05), but no difference in mean BMI was observed between Caucasian and non-Caucasian participants.

At baseline, no differences were observed with regard to the proportions of participants with diabetes and using diabetes medications. At baseline, the participants in the Dublin cohort reported more HTN (*p* < 0.05), fewer challenges in performing their daily activities (*p* < 0.01), and less anxiety and depression (*p* < 0.05) than the UK cohort. The patients in the Dublin cohort were also older and had lower BMIs (Table 2).

### 3.2. Weight-Related Outcomes at Week 52

Data were analysed from all participants with a full data set. Data from 93 participants were analysed at 52 weeks, with n = 27 from the Dublin cohort and n = 66 from the UK cohort (Table 3). At 1 year, 14.8% (4/27) of the Dublin participants and 3.0% (2/66) of the UK participants achieved >15% WL, representing a non-significant trend toward greater WL in the Dublin cohort (*p* = 0.07). Participants in the Dublin cohort lost more kilograms and a greater percentage of body weight at all time points. At week 52, a significantly higher proportion of participants from Dublin achieved ≥5% WL compared to the UK cohort (*p* < 0.05).

### 3.3. Weight-Related Outcomes at Week 104

Data were available for n = 61 participants at week 104, with n = 40 from the UK cohort and n = 21 from the Dublin cohort (Table 3). At this time point, participants in the Dublin cohort were significantly more likely to have lost ≥10% of their baseline body weight compared to their UK counterparts (*p* < 0.05). From baseline to week 104, participants in the UK cohort gained an average of 0.71 kg (SD ± 9.52), while those in the Dublin cohort lost an average of 5.7 kg (SD ± 10.88).

### 3.4. Changes in Parameters Relating to Metabolism

#### 3.4.1. Waist Circumference

Data were available for n = 61 participants at week 104, with n = 40 from the UK cohort and n = 21 from the Dublin cohort (Tabel 3). At each of these time points, there was no statistically significant difference in waist circumference between the Dublin and UK cohorts (all *p* = 0.09).

#### 3.4.2. Glycaemia

Data were available for n = 61 participants at week 104, with n = 40 from the UK cohort and n = 21 from the Dublin cohort (Tabel 3). At baseline, there was no difference in HbA1c between the Dublin and UK cohorts. However, at week 52, in the Dublin cohort, the HbA1c decreased from baseline, whereas in the UK cohort, it increased from baseline (*p* = 0.03). This difference became more pronounced at week 104, when the HbA1c increased from baseline in the UK cohort and further decreased in the Dublin cohort (*p* = 0.02).

### 3.5. Changes in Parameters Relating to Quality of Life

#### 3.5.1. Anxiety/Depression

Data were analysed for 46 participants from the UK cohort and 15 participants from the Dublin cohort. The participants in the Dublin cohort were less likely at baseline to report anxiety/depression on the EQ5D (*p* = 0.015). No difference was noted between cohorts for improvement in anxiety/depression self-reporting at week 52. At week 52, 29.5% of all the participants had worsening in their anxiety/depression self-rating between baseline and week 52, which was associated with weight gain from baseline. At week 104, 14.8% of all the participants had worsening in their anxiety/depression self-rating between the visits at weeks 52 and 104, which was also associated with weight gain from baseline.

#### 3.5.2. Ability to Perform Self-Care/Activities of Daily Living

Data were available for n = 46 from the UK cohort and n = 15 from the Dublin cohort. At baseline, there was no difference between the UK or Dublin cohorts for self-rated ability to perform activities of daily living. No difference was observed at week 52 or 104 between the cohorts.

#### 3.5.3. Ability to Carry out Usual Activities

Data were available for n = 46 participants from the UK cohort and n = 15 participants from the Dublin cohort. At baseline, there was no difference between the UK or Dublin cohorts in terms of the participants’ ability to carry out usual activities. No difference was observed at week 52 or 104 between cohorts. At week 52, 26.2% of all the participants reported a decline in their ability to carry out day-to-day tasks from baseline, which was associated with weight gain from baseline.

#### 3.5.4. Pain

Data were available for n = 45 from the UK cohort and n = 15 from the Dublin cohort. At baseline, there was no difference between UK or Dublin cohorts in self-rated pain/discomfort. No difference was observed at week 52 or 104 between cohorts. At week 52, 33.3% of all the participants had worsening in their self-reported pain/discomfort, which was associated with weight gain from baseline. A further 21.7% reported worsening from week 52 to 104, which was associated with more weight gain from week 52 to 104.

#### 3.5.5. Mobility

Data were available for n = 45 from the UK cohort and n = 15 from the Dublin cohort. At baseline, there was no difference between the UK and Dublin cohorts in self-rated mobility challenges. No difference was observed at week 52 or 104 between cohorts. At week 52, 30.0% of all the participants had worsening in their self-reported mobility, which was associated with weight gain from baseline. A further 16.7% reported deterioration from week 52 to 104. This was associated with weight gain from week 52 to 104.

### 3.6. Changes in Parameters Relating to Complications of Obesity

#### 3.6.1. Diabetes Onset

At weeks 52 and 104, no participants in the Dublin cohort developed new-onset diabetes, as defined by a HbA1c > 48 mmol/mol (Tabel 3). In the UK cohort, 3.3% (n = 3) developed new-onset diabetes at week 52, which was associated with a weight gain of 2.1% from baseline. At week 104, a further 3.3% (n = 3) from the UK cohort developed new-onset diabetes. This was associated with a weight gain of 3.2% between weeks 52 and 104.

#### 3.6.2. Cardiometabolic Risk Factors

From baseline, more participants in the Dublin cohort had HTN than in the UK cohort (*p* < 0.05), but there was no difference in the use of antihypertensive medications. The participants in the Dublin cohort had higher systolic (*p* = 0.01) and diastolic (*p* = 0.004) blood pressure at baseline. However, by week 52, there were no differences in the development or remission of HTN between cohorts.

## 4. Discussion

The Dublin cohort lost 4.9% more weight than the UK cohort. The reasons for this may partly relate to the use of meal replacements for breakfast and lunch as part of usual care in the Dublin cohort. Previous work from the Glasgow site on complete meal-replacement intervention also demonstrated a higher proportional WL with this intervention than with food-based caloric restriction [12].

Socioeconomic factors may also play an important role, as the majority of participants within the Dublin cohort were domiciled within commuting distance of the clinical trial site, which was situated in an affluent part of Dublin. Lower socioeconomic status has previously been shown to be a hindrance in adherence to lifestyle intervention programmes [13]. Furthermore, given the nature of a clinical trial, people of higher socioeconomic and educational status are more likely to be recruited and to display positive outcomes [14]. The trial sites within the UK had greater heterogeneity in terms of socioeconomic considerations given the variation in site locations.

Participants in the Dublin cohort were, on average, 5 years older than those in the UK cohort. Current evidence on the implications of age in WL is mixed and context-dependent. Older patients may have a greater response to lifestyle intervention [15]. One of the limitations of the use of WL as a measure of health gain in obesity care is that it does not encompass changes in muscle mass. With increasing age comes an increasing risk of sarcopenia, notably during periods of WL [16]. Thus, one of the limitations to the interpretation of WL in this older cohort is the inability to control for absolute body-composition changes beyond waist circumference. Protein intake, which can be increased with meal replacements, and strength training remain important protective factors for preventing muscle loss in sarcopenic obesity [17]. In addition to age and socioeconomic status, other baseline differences between the cohorts—such as lower BMIs and lower rates of self-reported anxiety or depression in the Dublin group—may have influenced the observed outcomes. These imbalances were not adjusted for in the analysis, and thus represent potential confounding factors. While the use of meal replacements likely contributed to the greater WL in the Dublin cohort, it is also possible that differences in baseline health status, psychological wellbeing, or readiness to engage in treatment played a role. These factors should be considered when interpreting the findings of this post hoc analysis.

### Secondary Outcomes

The Dublin cohort had greater improvements in glycaemic control at weeks 52 and 104 (*p* = 0.02). The greater WL is a possible explanation, especially as more patients achieved >10% WL in the Dublin cohort, which may be sufficient to disrupt type 2 diabetes, but also to prevent the development of type 2 diabetes [18].

The Dublin cohort had more HTN at baseline and at weeks 52 and 104, despite having more WL and no difference in medication use. This would suggest that WL achieved through a non-pharmacological approach has a minor effect on treating the disease of HTN. More WL may have been needed. Our study may be underpowered to detect the association between WL and improvement in blood pressure, because we had too few patients who achieved more than 10% WL within the standard-care arm.

Despite the greater WL in the Dublin participants, there were no differences during follow-up in anxiety, depression, ability to perform self-care, activities of daily living, ability to carry out usual activities, pain, and mobility. This may be a type II statistic error due to the sample size, or it may be due to the fact that the Dublin cohort started with a good baseline, thus making improvements less easy to observe.

An important limitation of this post hoc analysis is that the study design did not set out to compare different strategies for the standard-care arm. However, this is also a strength, because all centres used their own standard care, and hence it reflected real-world evidence. Even though there was a difference in the WL achieved, the effect size of the WL was not sufficient to result in major changes as regards the complications of obesity. The study was underpowered to show changes in HTN, type 2 diabetes, and quality of life measures, although the trends associated with greater WL in the Dublin cohort were consistent with the expected effect on obesity complications. The lack of more detailed body composition analysis was a major limitation, and this should be included in future studies.

In conclusion, while there were modest differences observed pre-intervention between the Dublin and UK cohorts, the Dublin cohort randomised to the standard-care arm did lose more weight than the UK cohort. The reasons for this may relate to variation in socioeconomic status and or the use of meal replacements. People living with obesity should be informed that standard lifestyle treatments with or without meal replacements remain a valid treatment option, but the percentage of patients who lose more than 15% body weight at 52 weeks or more than 10% bodyweight at 104 weeks remains below 20%.

## Figures and Tables

**Table 1:** Summary of categorical baseline characteristics.

Baseline Characteristic	Total Population (n = 132)		UK Cohort (n = 92)		Dublin Cohort (n = 40)		* p*-Value
	Number	Percentage	Number	Percentage	Number	Percentage	
Sex							
Men	53	40.2	35	38.0	18	45.0	0.454
Women	79	59.9	57	62.0	22	55.0	
Missing	0	0.0	0	0.0	0	0.0	
Ethnicity							
White	114	86.4	74	80.4	40	100.0	0.028
Black	11	8.3	11	12.0	0	0.0	
South Asian	3	2.3	3	3.3	0	0.0	
Mixed/Other	4	3.0	4	4.4	0	0.0	
Missing	0	0.0	0	0.0	0	0.0	
Glycaemic Status							
Normoglycaemia	66	50.0	41	44.6	25	62.5	0.184
Pre-diabetes	20	15.2	17	18.5	3	7.5	
Diabetes remission	3	2.3	3	3.3	0	0.0	
Diabetes	41	31.1	29	31.5	12	30.0	
Missing	2	1.5	2	2.2	0	0.0	
Hypertension status							
No	41	31.1	34	37.0	7	17.5	0.032
Yes	90	68.2	58	63.0	32	80.0	
Missing	1	0.8	0	0.0	1	2.5	
Number of diabetes medications							
Does not have diabetes	91	68.9	63	68.5	28	70.0	0.560
0	12	9.1	8	8.7	4	10.0	
1	22	16.7	17	18.5	5	12.5	
2	6	4.6	4	4.4	2	5.0	
≥3	1	0.8	0	0.0	1	2.5	
Number of antihypertensive medications							
Does not have hypertension	44	33.3	33	35.9	11	27.5	0.456
0	24	18.2	18	19.6	6	15.0	
1	25	18.9	16	17.4	9	22.5	
2	22	16.7	16	17.4	6	15.0	
≥3	17	12.9	9	9.8	8	20.0	
EQ5D—Mobility							
1	39	29.6	26	28.3	13	32.5	0.211
2	37	28.0	24	26.1	13	32.5	
3	32	24.2	25	27.2	7	17.5	
4	17	12.9	14	15.2	3	7.5	
5	1	0.8	1	1.1	0	0.0	
Missing	6	4.6	2	2.2	4	10.0	
EQ5D—Self-care							
1	89	67.4	61	66.3	28	70.0	0.073
2	18	13.6	13	14.1	5	12.5	
3	17	12.9	14	15.2	3	7.5	
4	3	2.3	3	3.3	0	0.0	
5	0	0.0	0	0.0	0	0.0	
Missing	5	3.8	1	1.1	4	10.0	
EQ5D—Usual activities							
1	49	37.1	31	33.7	18	45.0	0.008
2	40	30.3	29	31.5	11	27.5	
3	27	20.5	21	22.8	6	15.0	
4	12	9.1	11	12.0	1	2.5	
5	0	0.0	0	0.0	0	0.0	
Missing	4	3.0	0	0.0	4	10.0	
EQ5D—Pain/discomfort							
1	20	15.2	12	13.0	8	20.0	0.161
2	48	36.4	35	38.0	13	32.5	
3	32	24.2	21	22.8	11	27.5	
4	18	13.6	15	16.3	3	7.5	
5	8	6.1	7	7.6	1	2.5	
Missing	6	4.6	2	2.2	4	10.0	
EQ5D—Anxiety/depression							
1	57	43.2	37	40.2	20	50.0	0.015
2	40	30.3	30	32.6	10	25.0	
3	23	17.4	17	18.5	6	15.0	
4	6	4.6	6	6.5	0	0.0	
5	2	1.5	2	2.2	0	0.0	
Missing	4	3.0	0	0.0	4	10.0	

**Table 2:** Summary of continuous baseline characteristics.

Baseline Characteristic	Total Population (n = 132)					UK Cohort (n = 92)					Dublin Cohort (n = 40)					^ 1 ^* p*-Value
	N Missing	Mean	SD	Median	IQR	N Missing	Mean	SD	Median	IQR	N Missing	Mean	SD	Median	IQR	
Age, years	0	51.81	10.77	53.1	45.5, 58.8	0	50.15	10.26	51.0	44.5, 56.8	0	55.63	11.09	56.9	48.8, 65.4	0.008
Body mass index, kg/m^2^	1	45.52	7.25	43.7	40.5, 49.4	0	46.58	7.54	45.5	40.9, 50.0	1	43.02	5.89	41.4	40.0, 47.0	0.004
Waist circumference, cm	2	131.81	12.95	131.0	122.0, 142.3	0	132.22	13.36	131.4	123.2, 143.8	2	130.82	12.02	130.5	120.8, 140.0	0.480
HbA1c, mmol/mol	2	44.71	10.79	41.0	38.0, 47.0	2	45.07	10.29	42.0	39.0, 47.0	0	43.90	11.93	40.0	38.0, 47.5	0.097
HbA1c, %	2	6.24	0.99	5.9	5.6, 6.5	2	6.28	0.94	6.0	5.7, 6.5	0	6.16	1.09	5.8	5.6, 6.5	0.091
Systolic blood pressure, mmHg	1	138.12	17.79	137.0	124.0, 152.0	0	135.85	18.12	132.5	124.0, 146.8	1	143.49	15.95	145.0	131.0, 155.0	0.012
Diastolic blood pressure, mmHg	1	81.99	12.04	81.0	74.0, 90.0	0	80.20	11.39	79.5	74.0, 87.8	1	86.23	12.62	86.0	80.0, 96.0	0.004

^1.^ UK sites included Glasgow (n = 9), Leicester (n = 30), Liverpool (n = 26), and London (n = 27). Standard Deviation (SD). *p*-value compares mean value of “UK cohort” and “Dublin cohort”.

**Table 3:** Summary of primary and secondary outcomes at 52 weeks and 104 weeks.

Outcome Measure	UK Cohort		Dublin Cohort		* p*-Value
	Number	Percentage or (Standard Deviation)	Number	Percentage or (Standard Deviation)	
Outcomes at 52 weeks	n = 66		n = 27		
>15% weight loss	2	3.0%	4	14.8%	0.07
Waist circumference, cm	128.7	(11.7)	126.9	(12.1)	0.09
HbA1c, mmol/mol	47.1	(11.2)	41.9	(10.3)	0.03
New-onset diabetes	3		0		
Systolic blood pressure, mmHg	136.9	(17.1)	140.1	(14.9)	0.38
Diastolic blood pressure, mmHg	81.6	(13.8)	84.1	(12.9)	0.22
					
Outcomes at 104 weeks	n = 40		n = 21		
>10% weight loss	1	2.5%	3	14.3%	0.04
Waist circumference, cm	128.9	(14.1)	126.5	(13.4)	0.09
HbA1c, mmol/mol	47.4	(10.7)	40.7	(10.6)	0.02
New-onset diabetes	6		0		
Systolic blood pressure, mmHg	137.3	(18.4)	140.3	(15.2)	0.31
Diastolic blood pressure, mmHg	82.3	(13.1)	83.5	(13.7)	0.47

## Data Availability

The original contributions presented in this study are included in the article. Further inquiries can be directed to the corresponding author(s).
